# Assessing men with erectile dysfunction before and after living donor liver transplantation in real-world practice: Integrating laboratories into clinical settings

**DOI:** 10.1371/journal.pone.0206438

**Published:** 2018-11-20

**Authors:** Heng-Chieh Chiang, You-Chiuan Chien, Ping-Yi Lin, Hsiu-Ling Lee, Yao-Li Chen

**Affiliations:** 1 Division of Urology, Department of Surgery, Changhua Christian Hospital, Changhua, Taiwan; 2 Department of Chemical Engineering, Chung Yuan Christian University, Chungli, Taiwan; 3 Transplant Medicine & Surgery Research Center, Changhua Christian Hospital, Changhua, Taiwan; 4 School of Medicine, Kaohsiung Medical University, Kaohsiung, Taiwan; 5 Department of General Surgery, Changhua Christian Hospital, Changhua, Taiwan; University of Toledo, UNITED STATES

## Abstract

**Objective:**

To evaluate the predictive role of the Model for End-Stage Liver Disease (MELD) score concerning changes in testosterone levels following living donor liver transplantation (LDLT) and the effects of LDLT on total testosterone and sex hormone-binding globulin (SHBG) levels, the free androgen index (FAI) and erectile function in LDLT recipients.

**Participants:**

41 adult male recipients of LDLT were evaluated before transplantation and six months after LDLT.

**Main outcome measures:**

We evaluated the effects of LDLT on total testosterone and SHBG levels, the FAI and erectile function in LDLT recipients. In this prospective study, MELD score, serum total testosterone, SHBG levels and FAI were measured in the morning of the operation day and 1 month, 3 months and 6 months after LDLT. The 5-item version of the International Index of Erectile Function (IIEF-5) questionnaire was administered before LDLT and six months after LDLT to evaluate erectile function.

**Results:**

The main outcome measure was dynamic parameter changes of total testosterone, SHBG, FAI and erectile dysfunction. The mean FAI value before LDLT was 16.75±10.10. The mean FAI was significantly higher 1 month (32.75±15.56; p < 0.01), 3 months (25.23±10.26; p < 0.01) and 6 months (29.16±11.05; p < 0.01) after LDLT. Mean IIEF-5 scores significantly increased after LDLT (from 11.7±7.7 before LDLT to 14.7±7.5, p< 0.01).

**Conclusions:**

MELD score correlates with severity of hypogonadism in men with end-stage liver disease. LDLT results in a reduction in serum levels of SHBG, an increase in FAI and improvement in erectile function.

## Introduction

Liver transplantation is considered the only curative therapy for patients with end-stage liver disease (ESLD)[1]. The Model for End-Stage Liver Disease (MELD) is a scoring system for evaluating the severity of ESLD[2]. MELD was originally developed to predict mortality in patients who had undergone transjugular intrahepatic portosystemic shunt procedures, but it has also been shown to be a useful assessment of patients with ESLD. Patients with more serious conditions of ESLD often have higher MELD scores.

Hypogonadism is also a common clinical condition in patients with ESLD.[3] Males with hypogonadism may present with muscle wasting and the absence of secondary sex characteristics, ambition and energy. Many of the clinical features of advanced liver disease are similar to those exhibited by men with hypogonadism, including low libido, general weakness, high fatigue level, depression, testicular atrophy and impotence.[4] The total testosterone level is lower than normal in most men with liver disease, and the level tends to decrease markedly as the disease progresses. An observational study conducted by Grossmann et al. revealed that low total testosterone was an independent predictor of mortality in patients with chronic liver disease.[5] Free testosterone is not bound by sex hormone-binding globulin (SHBG). Therefore, free testosterone is able to pass through the cell membrane and considered as biologically active.

Changes of SHBG concentrations in the human body are the consequences of several diseases, and high values of SHBG are found in hyperthyroidism, hypogonadism, androgen insensitivity and hepatic cirrhosis in men.[6] Excessive SHBG levels, which are commonly detected in patients with ESLD, result in decreased levels of free testosterone and explain the clinical symptoms of hypogonadism in ESLD patients.[7–10] Several studies have demonstrated that orthotopic liver transplantation (OLT) results in normalization of total testosterone and SHBG levels, thereby ameliorating hypogonadism.[11,12] However, there is no study focusing on the impact of total testosterone and SHBG.

Erectile dysfunction (ED) is defined as the inability to attain or maintain an erection suitable for satisfactory sexual intercourse.[13] The etiologies of ED are multi-factorial, including vascular, neurogenic, psychogenic, and endocrine-related components.[14] ED is common in patients with ESLD.[15] Possible mechanisms of ED in patients with ESLD include dysfunction of the hypothalamus-pituitary-gonadal axis, changes in the estrogen-over-androgen ratio and alteration of SHBG.[14] We previously demonstrated that living donor liver transplantation (LDLT) results in improvement in erectile function and self-reported restoration of hypogonadism, which was measured by the Androgen Deficiency in the Aging Male questionnaire.[15]

Few studies have addressed the relationship between severity of ESLD and hypogonadism or assessed the impact of LDLT on hypogonadism in a dynamic and continuous fashion. Therefore, we conducted a prospective cohort study to further determine the impact of LDLT on total testosterone levels, SHBG and erectile function in men with ESLD.

## Materials and methods

### Patients

**Fig 1:**
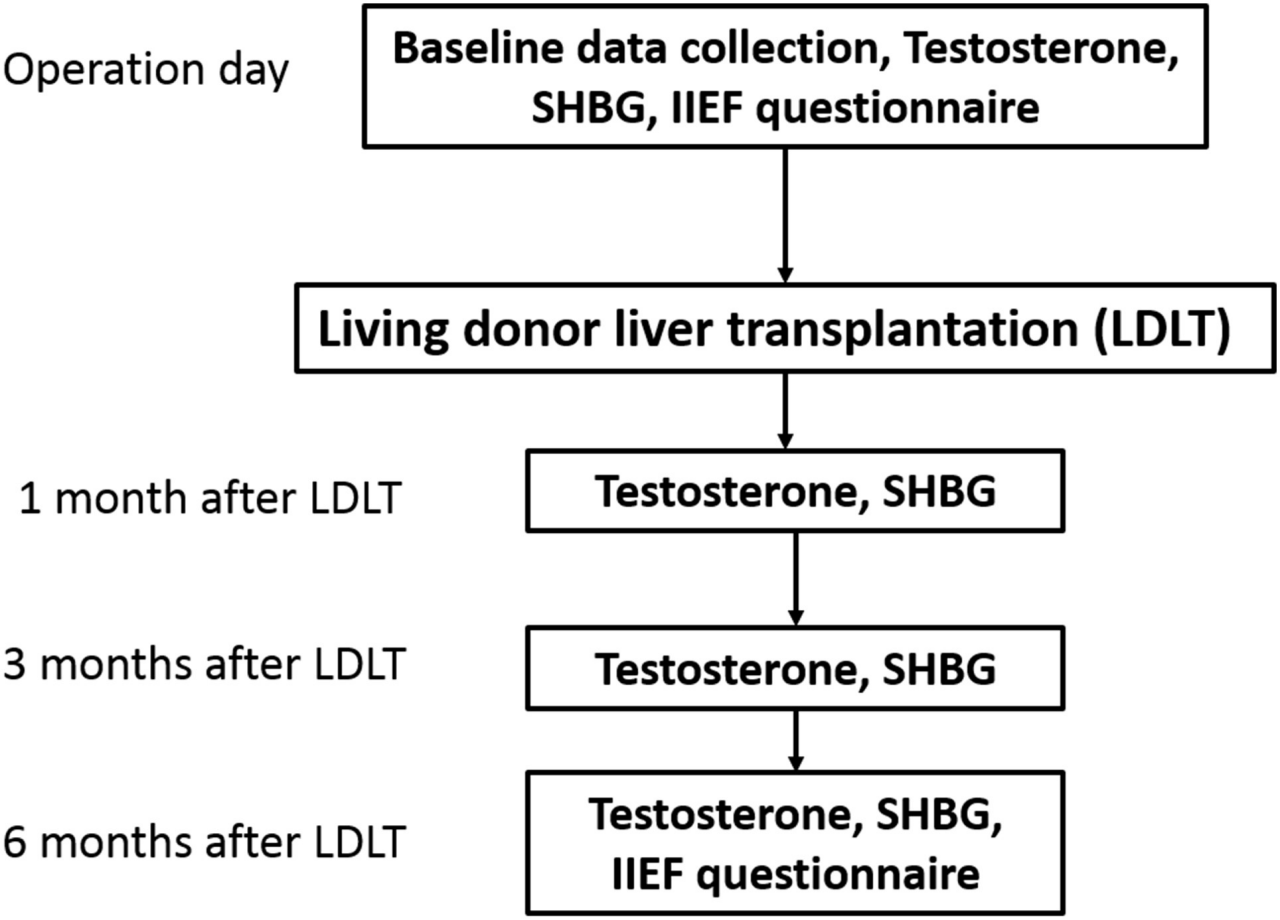
Flow chart of patient selection in the study.

The study protocol was approved by the Institutional Review Board of the Changhua Christian hospital and all patients provided written informed consent. None of the transplant donors were from a vulnerable population and all donors or next of kin provided written informed consent that was freely given. Patients in this prospective study comprised 41 men with ESLD who were scheduled to undergo LDLT at the Changhua Christian Hospital (Changhua, Taiwan) during the period 2013–2014 (Fig 1). An introductory letter containing a description of the methodology of the study, the possible results, contact information of the study team, and related information was presented to every participant before LDLT. Etiology of liver disease (hepatitis B virus, hepatitis C virus, or alcoholism), alcohol intake history, the presence of diabetes and demographic data were also collected (S1 File).

### Inclusion and exclusion criteria

Adult male recipients of LDLT were considered eligible for inclusion. Exclusion criteria included female gender, age less than 18 years, liver graft failure, refusal to complete the erectile function questionnaire and failure to follow-up for any reason.

### Survey

#### Main outcome measures

Hematologic and biochemical parameters were measured in-house using standard methods. For hormone assays, serum was centrifuged and then frozen in aliquots at −20°C until analysis occurred. According to Saad et al., the effects of testosterone therapy on sexual arousal appear after 3 weeks and plateau at 6 weeks; however, changes in erectile function take up to 6 months to occur.[16] Therefore, serum levels of total testosterone and SHBG were measured between 8 and 10 o’clock in the morning of the operation and 1 month, 3 months, and 6 months after LDLT. The level of total testosterone was measured in-house using a commercial immunoassay (ADVIA Centaur System, Bayer Health Care, Tarrytown, NY, USA) and an automated high-throughput immunology analyzer (ACS-180, Bayer Diagnostics, Puteaux, France). The normal reference range of total testosterone from 1.75 to 7.81 ng/ml with the functional sensitivity of 0.1 ng/ml was reported.

SHBG level was determined by an electrochemiluminescence immunoassay and analyzed by the cobas e411 immunoassay analyzer (Roche Diagnostics GmbH, Mannheim, Germany). The normal reference range for SHBG in men is 14.5–48.4 nmol/L. Free testosterone level was determined using the free androgen index (FAI) and the Vermeulen formula: FAI = 100 × testosterone / SHBG. The normal reference range of FAI is 15.5%-102.0%.[17] The MELD score was calculated using the United Network for Organ Sharing modification equation as follows: MELD Score = [0.957 × ln (serum creatinine) + 0.378 × ln (serum bilirubin) + 1.120 × ln (INR) + 0.643] × 10. There is no normal range for the MELD score, but a lower MELD score indicates a lower mortality rate in ESLD patients.

Medications for ED, including phosphodiesterase type 5 inhibitor, testosterone supplement, and prostaglandin E1, were not administered to patients before or after LDLT. The severity of ED was evaluated using the five-item International Index of Erectile Function (IIEF-5) questionnaire. Patients were asked to complete the IIEF-5 questionnaire before LDLT and six months after transplantation in an outpatient setting. The IIEF-5 questionnaire is a modified version of the International Index of Erectile Function (IIEF), a widely used tool to evaluate the severity of ED. The IIEF-5 questionnaire contained five questions, each scored from 1 point to 5 points. Therefore, the result of an IIEF-5 questionnaire is a score ranging from 5 to 25. The IIEF-5 questionnaire was designed to subjectively appraise the existence and severity of ED over a six-month period and has been shown to have a high sensitivity.[18] Severity of ED was classified into five categories according to IIEF-5 score: severe (score 5–7), moderate (score 8–11), mild-to-moderate (score 12–16), mild (score 17–21), and no ED (score 22–25).

For ESLD patients, the only known cure is liver transplant. Therefore, withholding a liver transplant from an ESLD patient is not ethical in research of human subjects. In our study, there is no so-called “control group”. Instead, we compared the baseline laboratory data and IIEF-5 scores with post-LDLT data to observe the effect of LDLT on the patients studied.

#### Statistical analysis

Categorical data were compared by the McNemar’s test or the Chi-square test. Differences in continuous variables were compared by the Wilcoxon signed-rank test. A *p* value of less than 0.05 was considered to indicate statistical significance. All statistical analyses were performed on a personal computer with the statistical package SPSS for Windows (Version 18.0, SPSS, Chicago, IL).

## Results

Of the 43 adult men who were eligible to participate in the study, 2 did not complete the questionnaires during the postoperative period because of privacy concerns. Table 1 summarizes the general clinical characteristics of the 41 patients who completed the study. The median age of the patients was 55 years and the median MELD score was 14. The etiologies of ESLD included alcoholic liver cirrhosis in 8 patients (19.5%), hepatitis B virus infection in 26 patients (63.4%) and hepatitis C virus infection in 7 patients (17.1%).

**Table 1:** Clinical baseline characteristics of the patients.

	Patients N = 41
	Mean ± SD
Age (years)	53.86 ± 7.53
Creatinine (mg/dL)	1.01 ± 0.51
Bilirubin (mg/dL)	4.26 ± 5.73
INR	1.45 ± 0.42
MELD score	14.59 ± 7.05
Total testosterone (pg/mL)	3.11 ± 1.95
Free testosterone (pg/mL)	16.75 ± 10.11
Sex hormone-binding globulin (nmol/L)	68.50 ± 35.99
Etiology of ESLD	(%)
Alcohol abuse	8 (19.5)
HBV	26 (63.4)
HCV	7 (17.1)
Erectile dysfunction category	
None	0 (0)
Mild	1 (2.4)
Mild-to-moderate	11 (26.8)
Moderate	11 (26.8)
Severe	18 (43.9)

INR = international normalized ratio; MELD = Model for End-Stage Liver Disease; ESLD = end-stage liver disease, HBV = hepatitis B virus; HCV = hepatitis C virus

### Changes in hormone profile after LDLT

**Fig 2:**
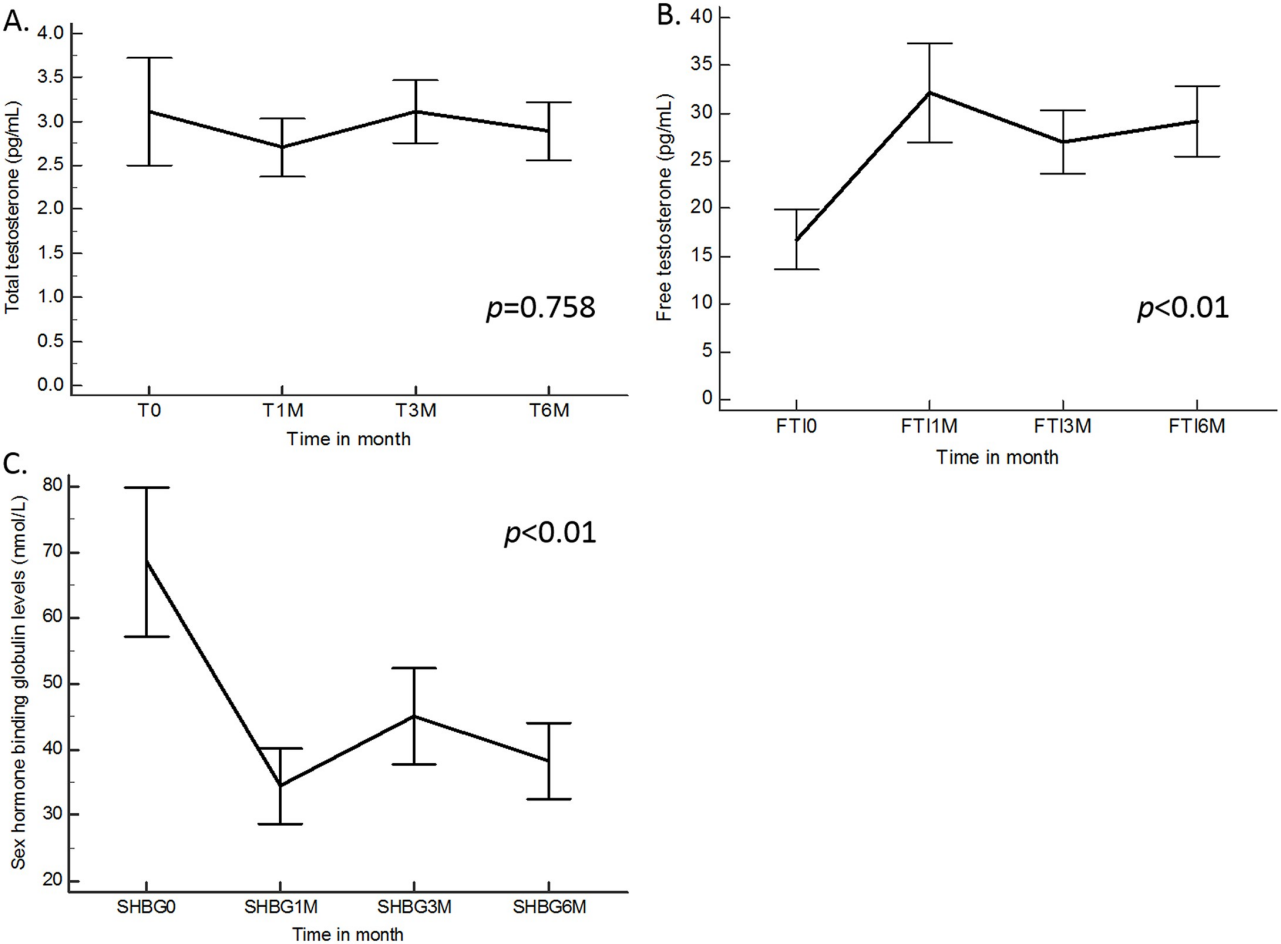
Changes of hormone profile. (A) Total testosterone (TT) levels, (B) Free testosterone (FT) levels and (C) Sex hormone-binding globulin (SHBG) levels at various times after LDLT.

There were no significant differences between the mean total testosterone level at baseline and the mean total testosterone level at any of the postoperative time points (*p* = 0.758). Follow-up measurements revealed that the mean SHBG level was significantly lower than baseline at 1 month (34.62±17.39 nmol/L; p < 0.01), at 3 months (44.74±23.53 nmol/L; *p* < 0.01) and at 6 months (38.23±17.01 nmol/L; *p* < 0.01) after LDLT. Mean FAI was significantly higher than baseline at 1 month (32.75±15.56; *p* < 0.01), at 3 months (25.23±10.26; *p* < 0.01) and at 6 months (29.16±11.05; *p* < 0.01) after LDLT (Fig 2).

**Fig 3:**
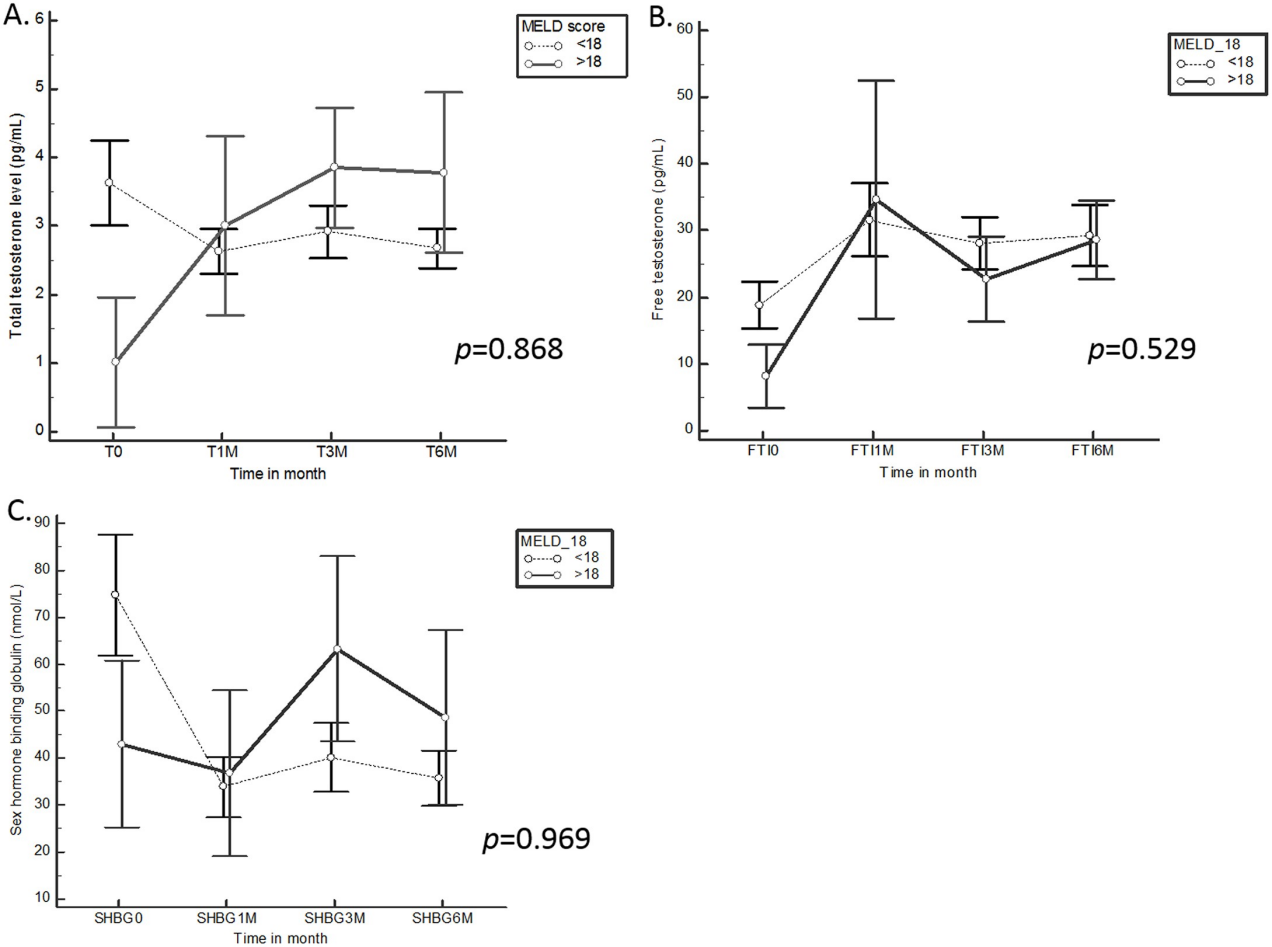
Correlation of MELD score and hormone profile. (A) Total testosterone (TT) levels, (B) Free testosterone (FT) levels and (C) Sex hormone-binding globulin (SHBG) levels according to MELD score (MELD below 18 versus MELD above 18).

We stratified patients into two MELD subgroups based on a MELD cutoff score of 18. There were no significant differences between the two groups of patients in changes in total testosterone, SHBG or FAI values after LDLT (Fig 3).

### Relationship between MELD score and sex hormone profile

MELD score negatively correlated with total testosterone and free testosterone levels prior to LDLT (r = -0.508 and -0.655 respectively). There was no relationship between MELD score and SHBG level before LDLT; however, MELD score correlated positively with SHBG level and IIEF-5 score after surgery. Prior to LDLT, total testosterone level significantly correlated with free testosterone and SHBG levels (r = 0.725 and 0.576, respectively). Free testosterone level before LDLT correlated with free testosterone and SHBG levels and IIEF-5 score after LDLT. IIEF-5 score prior to LDLT correlated with IIEF-5 score after LDLT. In addition, SHBG level after LDLT correlated with total testosterone and free testosterone levels after LDLT (Table 2).

**Table 2:** Correlation between MELD score, total testosterone, free testosterone and sex hormone-binding globulin.

	MELD score	1	2	3	4	5	6	7
1. Total testosterone (Before transplantation)	-.508**							
2. Free testosterone (Before transplantation)	-.655**	.725**						
3. Sex hormone-binding globulin (Before transplantation)	-.114	.576**	-.069					
4. IIEF-5 score (Before transplantation)	.117	-.030	.039	-.062				
5. Total testosterone (After transplantation)	.225	-.078	-.073	-.214	.096			
6. Free testosterone (After transplantation)	-.156	.268	.369*	-.151	.215	-.009		
7. Sex hormone-binding globulin (After transplantation)	.373*	-.304	-.396*	-.022	-.080	.548**	-.758**	
8. IIEF-5 score (After transplantation)	.432**	-.220	-.313*	-.025	.474**	.060	.264	-.040

*. Correlation is significant at the 0.05 level (2-tailed);

**. Correlation is significant at the 0.01 level (2-tailed).

### Change in erectile function

Over the six months following LDLT, the mean IIEF-5 score increased significantly from 8 before LDLT to 14 after LDLT (*p* <0.05), indicating that LDLT played at least a partial role in improving erectile function. We also found that most patients reported an improvement in ED category after LDLT (*p* <0.01) (Table 3).

**Table 3:** Erectile dysfunction categories before and after liver transplantation.

Categories based on IIEF-5 score	Before transplantation N (%)	After transplantation N (%)	*p* value
Severe ED	18 (43.9)	8 (19.5)	< 0.01
Moderate ED	11 (26.8)	8 (19.5)	
Mild-to-moderate ED	11 (26.8)	8 (19.5)	
Mild ED	1 (2.4)	14 (34.1)	
No ED	0 (0)	3 (7.3)	

IIEF-5 = five-item International Index of Erectile Function; ED = erectile dysfunction

### Correlation of MELD score, hormone profile and IIEF-5

**Fig 4:**
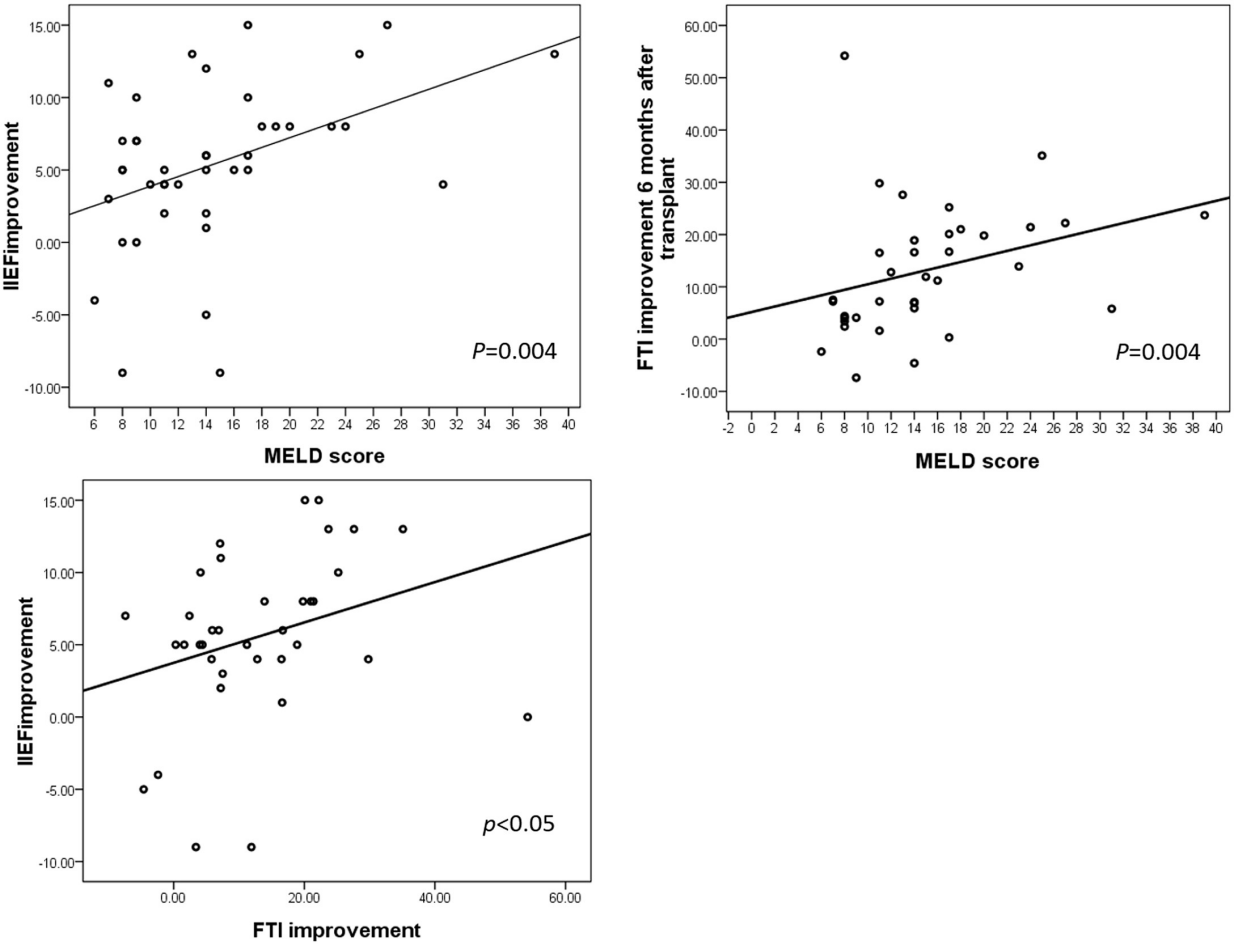
Correlation between Model for End-Stage Liver Disease (MELD) score, improvement of the five-item International Index of Erection Function -5 (IIEF-5) score and free androgen index (FAI).

MELD score correlated with improvement in FAI (*p*<0.01) and IIEF-5 score (*p*<0.01). In addition, improvement in FAI correlated with improvement in IIEF-5 (*p*<0.05) (Fig 4).

## Discussion

The MELD score has been shown to be a reliable means of predicting prognoses of patients with ESLD as well as a means for prioritizing recipients of liver transplants.[2,19,20] In our study, higher MELD scores correlated with lower total testosterone and free testosterone levels but not with lower SHBG levels before LDLT. This finding is consistent with Grossmann et al., who found that low testosterone was predictive of mortality in patients with chronic liver disease.[5] Interestingly, SHBG, which is a sensitive indicator of cirrhosis severity, was not shown to be a predictor of prognosis after liver transplantation surgery in our study.

Few studies have investigated the relationship between MELD score and sex hormone levels in conjunction with liver transplant. In a prospective study of 30 patients scheduled to undergo OLT, Nitsche et al. found that free T tended to increase after surgery in patients with MELD scores lower than 18, although there was no significant difference in postoperative free testosterone levels between patients with MELD scores below 18 and those with scores above 18.[7] In this study we also found no differences in testosterone levels after LDLT between patients with MELD scores above 18 and those with scores below 18, indicating that the MELD score is not predictive of hormonal outcome after liver transplantation.

Theoretically, ESLD causes a reduction in synthesis of liver proteins.[4] However, some literature addressed elevated SHBG levels in patients with ESLD, which is different from other proteins.[21] Sinclair et al. hypothesized that decreased total testosterone levels may result in elevated SHBG levels despite a reduction in protein synthesis in the liver.[4] In our study, high free testosterone levels correlated with high SHBG levels before LDLT, a finding similar to that reported by Burke et al., who found that excessive estrogen in patients with ESLD stimulated the production of SHBG.[22] Furthermore, in an observational study of 95 men with cirrhosis and 256 healthy control subjects, Sinclair et al. found that serum estrone level correlated well with MELD score and adverse outcome.[23] In a systematic review of 21 studies with a total population of 1274 patients with ESLD, Gariani et al. found decreased SHBG levels after liver transplantation in all four source studies and improvement of free testosterone in five cohort studies.[24] In our study, there was no significant change in testosterone level after LDLT. However, the SHBG levels were significantly lower after surgery, resulting in a significant increase in free testosterone levels. Moreover, the effect of LDLT on total and free testosterone levels was sustained for at least six months. Further clinical, genetic and molecular studies are needed to better understand the reasons why SHBG levels are increased in patients with ESLD.

Sexual dysfunction, particularly ED, is a common condition of a candidate for liver transplantation surgery.[25,26] The IIEF-5 and IIEF are the most widely used scales for measuring the severity of ED after orthotopic liver transplantation.[13,26–28] Chien et al. firstly used the IIEF-5 to assess ED in recipients of LDLT and reported improvement of ED in most of the patients studied.[15] All such studies concluded that liver transplantation plays a positive role in improving sexual function in patients with ESLD. Furthermore, a systematic review by Gariani et al. revealed that sexual performance improved after liver transplantation in 10 of the 11 studies reviewed.[24] In our study, there was significant improvement in erectile function after LDLT. In addition, improvement in free testosterone correlated with higher IIEF-5 scores after LDLT, suggesting that hormones play a role in the effect of LDLT on erectile function.

We acknowledge several limitations in this study. First, immunosuppressant usage after LDLT was not standardized. Therefore, pharmacologic factors could not be taken into account. Second, our findings are limited by the lack of sham procedures in the study. Third, the relatively short follow-up period precluded the assessment of the whole impact of LDLT on hormone and erectile function. Despite the above-mentioned limitations, this study is, to the best of our knowledge, the first to compare detailed changes in total testosterone, SHBG, free testosterone and ED after LDLT. Further large-scale, well-designed studies, however, are warranted to better understand the mechanisms underlying the positive effects of liver transplantation on sexual function.

## Conclusion

The MELD score correlates with severity of hypogonadism in men with ESLD. Living donor liver transplantation results in a reduction in serum level of SHBG, an increase in free testosterone and improvement in erectile function.

## Supporting information

S1 File (XLS)
